# Update on Febrile Neutropenia in Pediatric Oncological Patients Undergoing Chemotherapy

**DOI:** 10.3390/children8121086

**Published:** 2021-11-25

**Authors:** Federica Cennamo, Riccardo Masetti, Prisca Largo, Alberto Argentiero, Andrea Pession, Susanna Esposito

**Affiliations:** 1 Pediatric Clinic, Pietro Barilla Children’s Hospital, Department of Medicine and Surgery, University of Parma, Via Gramsci 14, 43126 Parma, Italy; fede.cennamo@gmail.com (F.C.); priscalar@live.it (P.L.); alberto.argentiero@unipr.it (A.A.); 2 Pediatric Oncology and Hematology Unit “Lalla Seragnoli”, Pediatric Unit-IRCCS Azienda Ospedaliero-Universitaria di Bologna, 40138 Bologna, Italy; riccardo.masetti5@unibo.it (R.M.); andrea.pession@unibo.it (A.P.)

**Keywords:** bacteremia, chemotherapy, febrile neutropenia, pediatric oncology, sepsis

## Abstract

Febrile neutropenia (FN) is a common complication of chemotherapy in oncological children and one of the most important causes of morbidity and mortality in these patients. The early detection of a bacteremia and the rapid therapeutic intervention are crucial to improve the outcome. We analyzed the literature in order to clarify the epidemiology of FN in children undergoing chemotherapy, the specific factors associated with a negative outcome, the most common etiology, and the value of biological markers as a tool to make an early diagnosis or to monitor the evolution of the infection. Several studies have tried to identify specific factors that could help the clinician in the detection of an infection and in its microbiological identification. However, due to the heterogenicity of the available studies, sufficient evidence is lacking to establish the role of these risk factors in clinical practice and future research on this topic appear mandatory. Determinations of risk factors, etiology, and markers of febrile episodes in these patients are complicated by the characteristics of the underlying illness and the effects of treatments received. Although some studies have tried to develop an evidence-based guideline for the empiric management of FN in pediatrics, validated predictive scores and algorithms are still lacking and urgently needed.

## 1. Introduction

Over the last decades, the prognosis of pediatric malignancies has progressively changed due to many factors, including a better knowledge of the biology of the diseases and an impressive improvement in supportive care. Nevertheless, febrile neutropenia (FN) remains a common complication of chemotherapy in oncological patients and one of the most important causes of morbidity and mortality [1,2]. Several researchers have tried to identify specific factors that could help the clinician in the detection of an infection and in its microbiological identification to improve the outcome of these children [2]. We analyzed the literature in order to clarify the epidemiology of FN in oncological children undergoing chemotherapy, the specific factors associated with a negative outcome in these patients, the most common etiology, and the value of biological markers as a tool to make an early diagnosis or to monitor the evolution of the infection.

## 2. Epidemiology of Febrile Neutropenia

FN is a leading cause of infectious mortality for oncological children receiving cytotoxic chemotherapies. Approximately one third of children treated for cancer or who underwent hematopoietic stem cell transplantation (HSCT) experienced FN during the neutropenic period [2]. Mortality associated with FN in these patients ranges from 2% to 6% [3]. The incidence and rate of febrile complications varies according to treatment intensity. In a prospective study, Castagnola et al. showed that the highest proportions of neutropenic periods with primary febrile episodes were observed after autologous HSCT (58%), aggressive treatment for acute leukemia (AL) or non-Hodgkin lymphoma (NHL, 48%), and allogeneic HSCT (44%); the lowest proportion (9%) was observed during maintenance chemotherapy for AL [2]. 

The most common causes of FN are bloodstream infections (BSIs). A large national study demonstrated that neutropenia was associated with BSIs in patients affected by AL in 84% of cases, in comparison with 47% of patients with solid tumor and 55% of patients who received bone marrow transplant [4]. Fever may also occur as part of the underlying diseases for a number of other causes, such as viral or fungal infections, drug or transfusion reactions or mucositis [5]. However, considering the importance of an early diagnosis in case of infectious etiology in order to decrease mortality [2], it is essential to consider pediatric oncological patients with FN as at high risk of infectious complications.

## 3. Predictive Factors for Sepsis Risk and Negative Outcomes in Children with Oncological Disease and Febrile Neutropenia

Several predictive factors have been evaluated in order to determine sepsis risk and negative in pediatric oncological patients. It has been demonstrated that previous episodes of FN and time from last chemotherapy ≤7 days increased the risk of BSI in febrile neutropenic children [6,7]. Bothra et al. observed that >3 previous FN episodes were associated with adverse outcomes such as mortality, invasive infections, and hemodynamic instability [7]. Rondinelli et al. reported that central venous catheter was an independent risk factor for severe infectious complications, including bacteremia in FN patients, particularly in the first phases of AL treatment [8]. Positive growth cultures of central line catheter during the previous 3 months increased the risk of bacteremia [6].

Neutropenia severity and duration relate directly to the development of sepsis in FN episodes, although the data from available studies are controversial. Kara et al. showed that neutrophil count <100/mm^3^ and lower white blood cell counts (WBC) were associated with bacteremia [6]. In addition, Rondinelli et al. observed that neutrophil count lower than 500 cells/mm^3^ represented a risk factor for severe complications in FN patients [8]. Freifeld and Pizzo observed that the risk to develop bacteremia and bacterial pneumonias were higher when neutrophil counts were <100 cells/mm^3^ [9]. They also reported that patients with long-term neutropenia were more susceptible to recurrent or new bacterial infections [9]. Similarly, Hughes et al. have demonstrated that neutrophil counts <100 cells/mm^3^ were associated with high incidence of bacteremia [9,10]. However, Regazzoni et al. found no association between neutrophil count at admission and mortality in these children [11]. In addition to neutropenia, the absolute monocyte count may be useful in identifying children at high risk for bacteremia [12]. Monocytes counts lower than 100 cells/mm^3^ represented an infection risk [8]. Madsen et al. showed that patients with high monocyte counts at admission presented a lower infection risk of bacteremia [13]. Other laboratory anomalies can represent a risk of infectious complications in FN pediatric patients, such as hemoglobin level <7 g/dL and platelet counts <20,000/mm^3^ [8]. Badiei et al. showed a significant association between a platelet count <20,000/mm^3^ and life-threatening infections [14]. Thrombocytopenia works as a marker for marrow suppression and increased consumption in sepsis. Das et al. pointed out that platelet count <20,000/mm^3^ was an additional predictor for infections, while albumin <2.5 g/dL and C reactive protein (CRP) >90 mg/L were risk factors for infection-associated mortality in FN patients [15]. CRP > 90 mg/dL is one of the strongest predictive factors of infectious complications in children with FN and this assumption was confirmed by several studies. Santolaya et al. reported that CRP >90 mg/L was associated with increased risk of invasive bacterial infection in children with cancer and FN [16]. An Indian research piece reported that CRP was useful to establish the diagnosis of infections, and serial CRP monitoring was necessary in order to evaluate the response to antibiotic therapy in children with FN [17]. Furthermore, Asturias et al. identified a direct association between elevated CRP levels and the duration of FN, bacteremia, and mortality [18]. Moreover, it was observed that serum lactate >3 mmol/L and serum bicarbonate <17 mmol/L were associated with septic shock and mortality in neutropenic children [19].

Some authors observed a worse outcome in malnourished patients with cancer [20]. Low albumin level predicted worse outcome in many diseases including FN because hypoalbuminemia is a marker for malnutrition and inflammatory state [15,21]. The risk of bacteremia appeared higher in neutropenic cancer patients with fever >39.0 °C [12,13,22]. Rondinelli et al. showed that temperature >38.5 °C in children with FN at admission was an independent predictive risk factor for septic shock [8]. Hypotension, tachycardia, and tachypnea were more often indicative of concurrent bacteremia in pediatric patients with FN. The National Institute for Health and Care Excellence (NICE) guidelines underline that hypotension and tachypnea were strong risk factors for septic complications [23]. In addition, Alberti et al. showed that heart rate >120/min and systolic blood pressure <110 mmHg were related to progression from sepsis to severe sepsis or septic shock [24]. 

The risk of sepsis in children with FN is different between hematological malignancies and solid tumors. Patients with hematological malignancies have higher risk for infection than patients with solid tumor. In fact, hematological diseases affect bone marrow and require more intensive myeloablative therapy, resulting in disruption of normal immune function [25]. Viscoli et al. observed that neutropenia was associated with bloodstream infections in patient affected by AL in 84% of cases, in comparison with 47% of patients with solid tumor and 55% of patients who received bone marrow transplant [4]. Furthermore, primary progressive or relapsed disease with bone marrow involvement in children <5 years old appeared predictive of septic complications [6].

The state of the disease and its treatment are related with sepsis predisposition. Haupt et al. reported that patients receiving intense chemotherapy had infection rates six times higher than those receiving less intense therapies [7]. Ammann et al. showed that bone marrow involvement in patients with cancer was associated with double risk of bacteremia compared to children with cancer without bone marrow infiltrations [26].

The site of infection is also correlated with infectious prognosis. Reilly et al. observed that upper airway infections were more frequent in children with FN than adults [27]. It was also observed that an identified focus predicted higher risk of serious complications [28].

Studies on a predictive score for sepsis risk and outcome in oncological children with FN were performed. Green et al. tested the Ammann score based on hemoglobin ≥9 g/dL, white blood cell count (WBC) <300/mm^3^, platelet count < 50,000/mm^3^ and intensive chemotherapy, although this score has not yet been prospectively validated [29]. In addition, Rondinelli et al. had suggested a score for predicting severe infection complications in patients with chemotherapy-induced FN and considered that severe bacterial infection was associated with bone marrow involvement, diagnosis of pre–B-cell leukemia, viral infection, CRP values, hemoglobin and leukocyte counts, and presence of central venous catheter [8]. Finally, Phillips et al. suggested the PICCNIC (predicting infections in children with cancer) predictive model was able to predict microbiologically documented infection: it included type of malignancy, maximum temperature, clinically severely unwell, hemoglobin, white cell count and absolute monocyte count [30]. 

Table 1 summarizes main risk factors associated with sepsis risk and negative outcomes in children with oncological disease and FN.

## 4. Etiology of Infections in Febrile Episodes

Over the last few decades, the etiology of microbial-related FN episodes has changed. In the 1960s and 1970s, the principal agents responsible of bacteremia in febrile neutropenia were Gram-negative bacteria (GNB), mainly *Escherichia coli*, *Klebsiella pneumoniae*, and *Pseudomonas aeruginosa*. In the 1980s bacteremia caused by Gram-positive cocci increased with the use of central line catheters, introduction of fluoroquinolone prophylaxis and use of intensive chemotherapy, causing severe mucositis [31]. However, bacteremia due to GNB increased again in the following years probably in correlation with the increase of fluoroquinolone-resistant GNB resulting from fluoroquinolone prophylaxis, and prevalence of extended-spectrum β-lactamase (ESBL)-producing *Enterobacteriaceae*, multidrug-resistant (MDR) *P. aeruginosa* and *Acinetobacter baumannii* [30]. Lee et al. observed that the proportion of GNB in bacteremia cases in children with FN increased in parallel with the developing of antibiotic-resistant (AR) [32]. They showed that AR GNB infections caused worse prognosis compared with non-AR bacterial infections [32]. In addition, Akova et al. reported that the incidence of Gram-positive bacterial infections raised with the use of central venous catheters, quinolone prophylaxis and broad-spectrum empirical antibacterial therapy, although Gram-negative pathogens remained predominant [33]. Oberoi et al. pointed out that *Staphylococcus aureus* and *E. coli* were the most common Gram-positive and Gram-negative identified bacteria in children with febrile neutropenia, respectively [34]. Instead, Jeddi et al. found that *K. pneumoniae* and *S. aureus* were the predominant Gram-negative and Gram-positive bacteria, respectively [35].

Currently, the rate of GNB in pediatric onco-hematological patients with FN is increasing, and *E. coli* is the predominant pathogen [36]. In the last years, the emergence and diffusion of MDR *E. coli* have been observed. In a trial performed in children with onco-hematological disorders, Wang et al. reported that the frequency of septic shock was 51.1%, higher than the average rate of 5–30% in studies of patients of all ages [37]. Despite the advent of potent antibiotics, *P. aeruginosa* infection is one of the most serious nosocomial infections related with high mortality in patients with immunosuppression or comorbidities, including FN [38,39]. 

Although bacteria are the most common agents involved in children with FN, fungal infections are an emerging concern. According to several studies, incidence of invasive fungal infection (IFI) varies from 2 to 36.5% [7,40]. Kumar et al. showed that Aspergillus sp. was the most common fungal isolate, followed by *Candida* species [41]. Accordingly, Lehrnbecher et al. observed that *Aspergillus* was the most common species of fungi followed by Candida in children with FN [42]. On the contrary, Gupta et al. [40] and Villarroel et al. [43] showed that *Candida* was the most frequently isolated fungal species from blood cultures in children with FN. Lai et al. showed that prolonged neutropenia >30 days, prolonged steroids therapy, relapsed malignancy and bone marrow transplant were significantly associated with IFI risk [44]. Moreover, Villarroel et al. showed found that hypotension or shock within 24 h, fever, Absolute monocyte count (AMC) <100/mm^3^ and C reactive protein CRP >90 mg/L were significant risk factors for IFI [43]. Several studies reported that death by fungal infection is relevant and deserves early intervention for prevention as well as treatment in pediatric FN [40,42,45]. 

The etiological diagnosis of FN in patients differ from those described in adults. A prospective multicenter study documented that in many FN episodes (78%), no microbiologically defined infection (MDI) was detected [46]. In addition, lack of MDI detection was related to shorter duration of fever and hospitalization, less need of intensive care and less intensive antimicrobial therapy compared with FN episodes with MDI [46]. The concept that infections can be observed only in a minority of pediatric cancer patients with FN has been challenged by studies relying on systematic identification of viral infections by molecular methods. In another two research studies, MDIs were reported in 67% and 60% of FN episodes, with respiratory virus infections detected by molecular methods in 57% and 46% of FN episodes, respectively [47]. The difference of MDI frequency is explained by different diagnostic procedures used for the detection of respiratory virus infections or by prevalence of non-infectious fever in these patients. 

## 5. Markers of Infections in Febrile Neutropenia

Early diagnosis of patients at low risk of infections in FN allows to avoid antibiotics reducing cost as well as antibiotic resistance, whereas the precocious detection of children at high risk permits a prompt antimicrobial therapy that permits to reduce complications and mortality [2]. Neutropenia significantly changes the inflammatory response of the host and several markers have been evaluated as markers of infections. Many cytokines have been studied to identify a marker which could stratify infectious risk in children with FN, but their role is still evolving. The most studied markers are procalcitonin (PCT), CRP, interleukin (IL)-6, IL-8, and IL-10, which are usually involved in the inflammatory mechanisms [48,49,50,51]. Interestingly, lactate was not explored as a diagnostic biomarker, although it represents a risk factor for septic shock and mortality [52].

PCT has a higher specificity for bacterial infections than other acute phase reactants and it is produced in multiple organ tissues during infection [53], with no influence caused by an alteration in the number and function of leukocytes. It was found that at diagnosis and also at the beginning of neutropenia, PCT concentrations were similar to basal levels in healthy controls, whereas CRP concentrations were moderately elevated [53]. Nevertheless, both PCT and CRP were significantly higher at fever onset although, in the case of CRP this increase was independent of the etiology of fever [53]. Fleischhack et al. showed that PCT was superior to CRP, IL-8 and IL-1β for distinguishing between bacterial and viral infections among patients with AL on the first day of FN [54]. They also observed that at admission PCT level could predict FN outcome [54].

Cytokines resulted to be less specific in detecting bacterial infections despite the precocity of their elevation. IL-6 and IL-8 have been shown to increase much earlier than CRP [55]. Narendra et al. demonstrated that CRP, IL-6, and IL-8 at admission were not useful to differentiate the infectious etiology in FN, but they confirmed the importance of rise in CRP and not of IL-6 or IL-8 in monitoring the response to treatment [56]. 

Regarding the specific etiology, Ruokonen et al. found that PCT has a poor sensitivity in patients with FN and Gram-positive infections [57]. In IFI, the role of PCT is still debated. One research study that analyzed recent literature, concluded that in the early phase of IFI PCT was elevated in fewer than half of invasive candidiasis episodes and in only one patient with invasive aspergillosis [58]. In addition, the role of serum PCT as a marker of prognosis in IFI has not been clarified. Studies of PCT levels in IFI have included a limited number of patients and the results are contradictory [59]. Christofilopoulou et al. found a significant PCT peak at around Day 10 associated with clinical complications [60]. This observation means that, according to their findings, the diagnostic role of PCT in IFI at onset is of limited value.

## 6. Conclusions

FN is a relevant cause of morbidity and mortality in pediatric oncological patients receiving chemotherapy. Early detection of an infectious etiology and the rapid therapeutic intervention are crucial to improve the outcome of these children. Several researchers have tried to identify specific factors that could help the clinician in the detection of an infection and in its microbiological identification. However, due to the heterogenicity of the available studies, sufficient evidence is lacking to establish the role of these risk factors in clinical practice and future research on this topic appear mandatory. Determinations of risk factors, etiology, and markers of febrile episodes in these patients are complicated by the characteristics of the underlying illness and the effects of treatments received. Although some studies have tried to develop an evidence-based guideline for the empiric management of FN in pediatrics [61,62,63], validated predictive scores and algorithms are still lacking and urgently needed.

## Figures and Tables

**Table 1:** Main risk factors associated with sepsis risk and negative outcomes in children with oncological disease and febrile neutropenia (FN).

Data	Negative Risk Factor
Clinical history	Previous history of FN
	Time from last chemotherapy ≤7 days
	Central venous catheter
Laboratory exams	Neutropenia severity (<100/mm^3^) and duration
	Monocyte count <100/mm^3^
	Hemoglobin level <7 g/dL
	Platelet count <20,000/mm^3^
	Albumin <2.5 g/dL
	C reactive protein >90 mg/L
	Serum lactate >3 mmol/L
	Serum bicarbonate <17 mmol/L
Clinical data	Malnutrition
	Fever >39 °C
	Hypotension
	Tachycardia
	Tachypnea
Type of cancer	Hematological malignancies
	Bone marrow involvement
	Requirement of intensive chemotherapy
	Bone marrow transplantation

## Data Availability

Not applicable in a review article.
